# A Systematic Review and Meta-Analysis of 3D Printing Technology for the Treatment of Acetabular Fractures

**DOI:** 10.1155/2021/5018791

**Published:** 2021-08-17

**Authors:** Jin Cao, Huanye Zhu, Chao Gao

**Affiliations:** Department of Orthopedics, Ningbo No. 6 Hospital, Ningbo, China

## Abstract

**Purpose:**

Three-dimensional (3D) printing technology has been widely used in orthopedics surgery. However, its efficacy in acetabular fractures remains unclear. The aim of this systematic review and meta-analysis was to examine the effect of using 3D printing technology in the surgery for acetabular fractures.

**Methods:**

The systematic review was performed following the PRISMA guidelines. Four major electronic databases were searched (inception to February 2021). Studies were screened using a priori criteria. Data from each study were extracted by two independent reviewers and organized using a standardized table. Data were pooled and presented in forest plots.

**Results:**

Thirteen studies were included in the final analysis. Four were prospective randomized trials, and nine used a retrospective comparative design. The patients aged between 32.1 (SD 14.6) years and 51.9 (SD 18.9) years. Based on the pooled analyses, overall, 3D printing-assisted surgery decreased operation time by 38.8 minutes (95% CI: -54.9, -22.8), intraoperative blood loss by 259.7 ml (95% CI: -394.6, -124.9), instrumentation time by 34.1 minutes (95% CI: -49.0, -19.1). Traditional surgery was less likely to achieve good/excellent function of hip (RR, 0.53; 95% CI: 0.34, 0.82) and more likely to have complications than 3D printing-assisted surgery (RR, 1.19; 95% CI: 1.07, 1.33).

**Conclusions:**

3D printing technology demonstrated efficacy in the treatment of acetabular fractures. It may improve surgery-related and clinical outcomes. More prospective studies using a rigorous design (e.g., randomized trial with blinding) are warranted to confirm the long-term effects of 3D printing technology in orthopedics surgeries.

## 1. Introduction

Acetabular fractures are usually caused by high-energy trauma in young adults or body-height falls in older adults [1]. There has been an increase in the incidence of acetabular fractures from 3.7 cases in 2006 to 5.0 cases in 2016 per 100,000 cases; in older adults aged over 75 years, the incidence was much higher, ranging from 17.1 to 23.2 cases [2]. Although relatively uncommon, acetabular fractures have been related to significant morbidity and mortality [3]. There have been significant advances in the treatment of acetabular fractures; however, this type of fracture remains one of the most challenging fractures to treat [4]. Acetabular fractures typically come with different fracture patterns, and there are numerous vascular and nervous elements surrounding the fractured areas [5]. The curved surface of the acetabulum makes the treatment very difficult. Because parts of the bone can only be touched without visualization during the operation, treating acetabular fractures requires exceptional visual and tactile skills. In addition to those skills, its treatment also requires a deep understanding of surgical anatomy. A slight incongruent reduction would result in postoperative osteoarthritis requiring total hip arthroplasty [6].

With the development of modern technology, new approaches, such as 3D printing, have opened a new era for the treatment of fractures [7]. The application of 3D printing technology varies from anatomical models mainly intended for preoperative planning to surgical guides and implants [8, 9]. 3D imaging can be used to assist CT scans to accurately visualize the fracture patterns [10, 11] and reduce the surgical margin of error [12]. Preoperative planning is a critical procedure of acetabular surgery and can also be assisted with 3D printing technology. After reduction, the plates should be precisely contoured in all three planes to fit the bone pelvis [13]. With the help of 3D printing technology, the pelvis could be turned around freely to simulate the surgical approach. The CT scan of the uninjured half of the pelvis could produce a mirror image that could be used to generate a 3D model of the acetabulum [14]. An even more advanced approach is the use of 3D printing technologies to create individualized implants [15–17]. This personalized regimen represents novel applications of 3D printing technology towards the trend of individualized patient care [18]. 3D printing technology also demonstrated extra values for complex cases [19–21].

It has been suggested that spine surgeons had a high interest in the incorporation of 3D printing technology into clinical practice [22, 23]. 3D printing technology also showed promising results in orthopedics surgeries. Recent evidence supports the feasibility of using 3D spinal implants [24]. Based on a systematic review of studies focusing on tibial plateau fractures, compared with conventional surgery, 3D printing technology-assisted surgery resulted in less operation time, intraoperative blood loss, and bony union time, without causing significant complications [25]. Similarly, 3D printing was found effective and safe in the surgical treatment of anatomically complex appendicular skeleton fractures [26]. In recent years, 3D printing technology is also used in the treatment of acetabular fractures. Nonetheless, whether patients could benefit from it remains unknown, warranting a comprehensive synthesis of current findings. Therefore, the aim of this systematic review and meta-analysis was to examine the efficacy of 3D printing technology for the treatment of acetabular fractures. Findings from this review may provide further evidence for more effective management of acetabular fractures and thereby improve the clinical outcomes of the patients.

## 2. Materials and Methods

A systematic review and meta-analysis were conducted to examine the effect of 3D printing technology in the treatment of acetabular fractures. This review was developed and reported following the Preferred Reporting Items for Systematic Reviews and Meta-Analyses (PRISMA) guidelines [27].

### 2.1. Search Strategy

A systematic search was conducted in PubMed, Web of Science, Embase, and Cochrane from inception to February 2021. The search was restricted to the English language. We used the combination of the following two sets of terms: (1) “3D,” “3-D,” “3 dimensional,” “3-dimensional,” “three dimensional,” or “three-dimensional”; AND (2) “acetabular fracture∗” or “acetabulum fracture∗.” The search terms were used in the title/abstract/keywords or subject terms. The bibliographies of eligible studies and previous reviews were reviewed to identify additional studies.

### 2.2. Eligibility Criteria

Studies that used 3D printing in the treatment of acetabular fractures were screened for eligibility. The inclusion criteria were as follows: (1) studies used a prospective or retrospective comparative design and (2) studies used 3D printing for acetabular fractures. The exclusion criteria were as follows: (1) studies that did not use 3D printing models or used them for education, simulation, and biomechanical testing purposes where no clinical outcomes were reported; (2) studies that did not have a control group or had an incomparable control group where the effect of 3D printing technology could not be evaluated; (3) nonhuman studies; (4) duplicated reports; and (5) other types of papers (e.g., case report or series, review, study protocol, abstract, or non-English).

### 2.3. Study Selection

Two independent reviewers screened the studies following the PRISMA flowchart [27]. In detail, firstly, the title/abstract of the retrieved studies was screened. Secondly, full texts of the potential studies were retrieved and reviewed. Lastly, the two reviewers determined the final inclusion based on the inclusion and exclusion criteria. A third reviewer was consulted in case of any discrepancy.

### 2.4. Data Extraction

Two independent reviewers extracted the data from each study. An extraction protocol describing which data to extract was developed by the team to facilitate the process. The protocol was piloted tested. In this review, we extracted characteristics of each study (e.g., first author, year of publication, country, and study design) as well as characteristics of the patients (e.g., sex, age, BMI, and disease duration). We also extracted the key outcomes and their assessment methods from each study including intraoperative and clinical outcomes (e.g., operation time, blood loss, instrumentation time, quality of reduction, hip function, and complications). For categorical variables, frequency and percentage were extracted. Mean and standard deviation (SD) were extracted for continuous variables, and frequency and percent were extracted for categorical variables. In the case of any discrepancy, a third reviewer was consulted.

### 2.5. Risk of Bias Assessment

The risk of bias was assessed by two independent reviewers. The Cochrane Risk of Bias Tool [28] was used to assess the risk of bias of RCTs. When using this tool, the following four aspects were assessed: performance bias, detection bias, attrition bias, and reporting bias. The quality of retrospective comparative studies was assessed using the Newcastle-Ottawa Scale (NOS) [29]. NOS evaluates three aspects including Selection, Comparability, and Exposure (in a case-control study). A maximum of two stars can be given for Comparability and one star for each numbered item within the Selection (four stars maximum) and Exposure (three stars maximum) categories. Adding scores from the three categories results in a total score, with higher scores indicating higher quality.

### 2.6. Statistical Analysis

Stata 12.0 (StataCorp LP, College Station, Texas) was used for data entry, management, and statistical analyses. For studies with missing data (e.g., SD), methods introduced in the Cochrane handbook were used to compute the data needed for the analysis. For instance, SD was calculated from the standard error of the mean or 95% confidence interval (95% CI). For continuous variables, the inverse variance approach was used to get a pooled weighted or standardized mean difference (WMD or SMD) with 95% CI. For categorical variables, the Mantel-Haenszel method was used to get a pooled risk ratio (RR) with 95% CI. Findings from each study and the pooled results were presented in forest plots. Subgroup analysis was conducted based on study design. The random effects model could account for unexplained heterogeneity by allowing the true effects underlying the studies to differ. This approach is suggested to be a more natural choice than the fixed effects model in medical research and thus was used in this review [30, 31]. Sensitivity analysis was conducted by the leave-one-out approach. Funnel plot for publication bias was not performed because of the small number of studies [32]. A *p* < 0.05 indicates statistical significance.

## 3. Results

**Figure 1:**
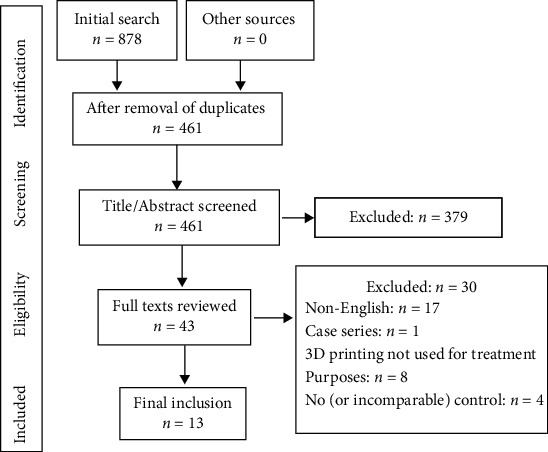
PRISMA flowchart for the process of study selection.

The study selection process is presented in Figure 1. A total of 878 records were identified through a systematic search of the databases. After removing duplications, 461 records were reviewed by the authors through reading the title and abstract. In total, 43 studies were retrieved and 30 were excluded after reading the full texts. Detailed exclusion criteria are listed in Figure 1. A total of 13 studies [33–45] were included in this review.

### 3.1. Risk of Bias

Detailed risk of bias assessment is presented in Table 1.  Table 1(a) presents the risk of bias in RCTs. The four trials were rated a low risk of bias on randomization, but it was unclear whether allocation concealment was used. Detection bias was rated low as the outcome measures were mainly objective and unlikely to be influenced by the knowledge of the intervention allocation.  Table 1(b) shows the quality of the nine retrospective comparative studies, with a NOS score of 8 (out of 10).

### 3.2. Study Characteristics

Characteristics of the 13 studies are shown in Table 2. The studies were published between 2018 and 2020 and were mainly conducted in China (*n* = 8). Two studies from the same research team were conducted in India [39, 40]. Four studies were prospective RCT, and nine used a retrospective comparative design. The sample size in each study ranged from 7 to 48 in the case group and 9 to 48 in the control group. Most of the studies used 3D printing technology for preoperative planning (e.g., precontoured plates). Two studies used 3D-printed plates during the surgery. Table 2 also shows the image processing and printing software used by each study as well as the key outcomes and measures.

### 3.3. Patient Characteristics

Characteristics of the patients are shown in Table 2. The patients had simple acetabular fractures or complex acetabular fractures. Time from injury to surgery was used as an inclusion or exclusion criteria in several studies, typically less than 2 or 3 weeks. Patients in the case and control group were overall comparable in age, sex, time from injury to surgery, and other indicators wherever reported. In brief, the patients aged between 32.1 (SD 14.6) years and 51.9 (SD 18.9) years. The patients were mainly males in most of the studies, except in one where all were male patients.

### 3.4. Effect of 3D Printing for Acetabular Fractures

The effect of 3D printing technology used for the treatment of acetabular fractures is shown in Table 3. Various surgery-related and clinical outcomes were assessed. Data across studies were quantified using meta-analyses.

#### 3.4.1. Operation Time

**Figure 2:**
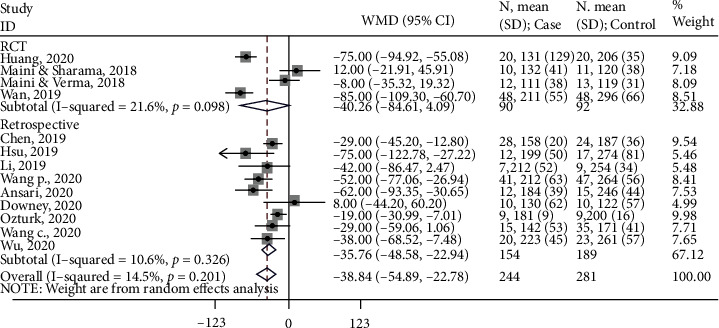
Forest lot for operation time. Subgroup-RCT: WMD (95%CI) = −40.3 (-84.6, 4.1) minutes, *z* = 1.78, *p* = 0.075; subgroup-retrospective: WMD (95%CI) = −35.8 (-48.6, -22.9) minutes, *z* = 5.47, *p* < 0.001; overall: WMD (95%CI) = −38.8 (-54.9, -22.8) minutes, *z* = 4.743, *p* < 0.001.

All 13 studies assessed operation time and were included in the meta-analysis. Based on the forest plot (Figure 2), overall, using 3D printing technology resulted in 38.8 minutes (95% CI: -54.9, -22.8) less operation time than the conventional method. Based on the subgroup analysis, when only findings from RCTs were included, 3D printing resulted in 40.3 minutes (95% CI: -84.6, 4.1) less operation time than the conventional method. Similarly, when findings from retrospective studies were included, 3D printing resulted in 35.8 minutes (95% CI: -48.6, -22.9) less operation time than the control group.

#### 3.4.2. Intraoperative Blood Loss

**Figure 3:**
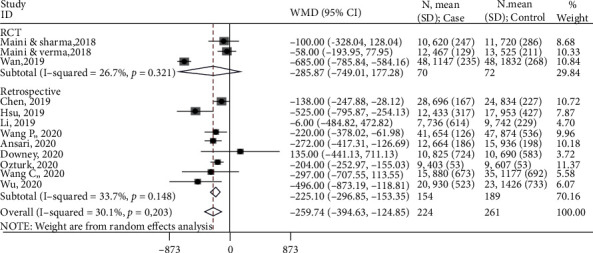
Forest lot for intraoperative blood loss. Subgroup-RCT: WMD (95%CI) = −285.9 (-749.0, 177.3) ml, *z* = 1.21, *p* = 0.226; subgroup-retrospective: WMD (95%CI) = −225.1 (-296.9, -153.3) ml, *z* = 6.15, *p* < 0.001; overall: WMD (95%CI) = −259.7 (-394.6, -124.9) ml, *z* = 3.77, *p* < 0.001.

All 13 studies measured intraoperative blood loss. One study [37] provided median and interquartile range. This study was not included in the meta-analysis. Thus, findings from 12 studies were included in the synthesis. Based on the forest plot (Figure 3), overall, 3D printing resulted in 259.7 ml (95% CI: -394.6, -124.9) less blood loss than the conventional method. Based on the subgroup analysis, when only findings from RCTs were included, 3D printing resulted in 285.9 ml (95% CI: -749.0, 177.3) less blood loss than the conventional method. Similarly, when findings from retrospective studies were included, 3D printing resulted in 225.1 ml (95% CI: -296.9, -153.3) less blood loss than the conventional method.

#### 3.4.3. Quality of Reduction

**Figure 4:**
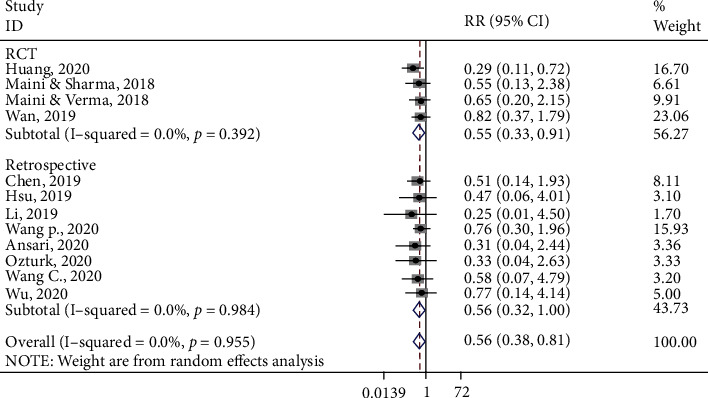
Forest plot for quality of reduction. Subgroup-RCT: RR (95%CI) = 0.55 (0.33, 0.91), *z* = 2.34, *p* = 0.019; subgroup-retrospective: RR (95%CI) = 0.57 (0.32, 0.98), *z* = 1.99, *p* = 0.045; overall: RR (95%CI) = 0.56 (0.38, 0.81), *z* = 3.06, *p* = 0.002.

Twelve studies assessed the quality of reduction and were included in the meta-analysis. Based on the forest plot (Figure 4), overall, the control group was less likely to achieve good/excellent reduction than the 3D printing group (RR, 0.55; 95% CI, 0.38, 0.81). Based on the subgroup analysis, when only findings from RCTs were included, the control group was less likely to achieve good/excellent reduction than the 3D printing group (RR: 0.55; 95% CI: 0.33, 0.91). Similarly, when findings from retrospective studies were included, the control group was less likely to achieve good/excellent reduction than the 3D printing group (RR: 0.57; 95% CI: 0.32, 0.98).

#### 3.4.4. Other Clinical Outcomes

**Figure 5:**
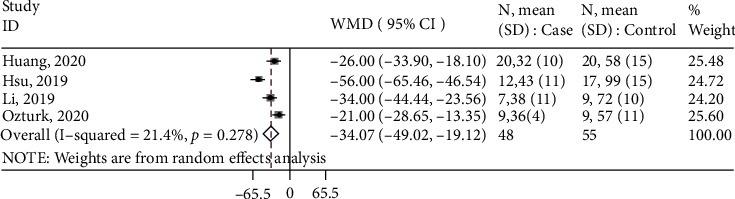
Forest plot for instrumentation time. Overall: WMD (95%CI) = −34.1 (-49.0, -19.1) minutes, *z* = 4.47, *p* < 0.001.

Other clinical outcomes included instrumentation time, function of the hip joint, and number/time of intraoperative fluoroscopy. Specifically, four studies measured instrumentation time and were included in the meta-analysis. Based on the forest plot (Figure 5), overall, 3D printing resulted in 34.1 minutes (95% CI: -49.0, -19.1) less instrumentation time than the conventional method.

**Figure 6:**
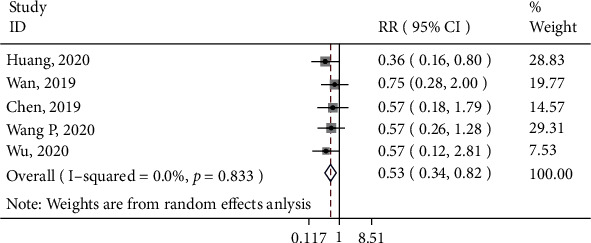
Forest plot for function of hip joint. Overall: RR (95%CI) = 0.53 (0.34, 0.82), *z* = 2.88, *p* = 0.004.

Six studies measured the function of the hip joint. One [33] reported the mean score instead of the percentage of good/excellent function. Thus, five studies were included in the meta-analysis. Based on the forest plot (Figure 6), overall, the control group was less likely to achieve good/excellent function of the hip than the 3D printing group (RR: 0.53; 95% CI: 0.34, 0.82).

Five studies measured the number or time of intraoperative fluoroscopy. Due to significant heterogeneity between the studies, data were not meta-analyzed. Overall, four studies found a significantly lower number or time of intraoperative fluoroscopy in the 3D printing group than in the control group. In one study [35], there was no significant difference in radiation exposure during the surgery.

#### 3.4.5. Complications

**Figure 7:**
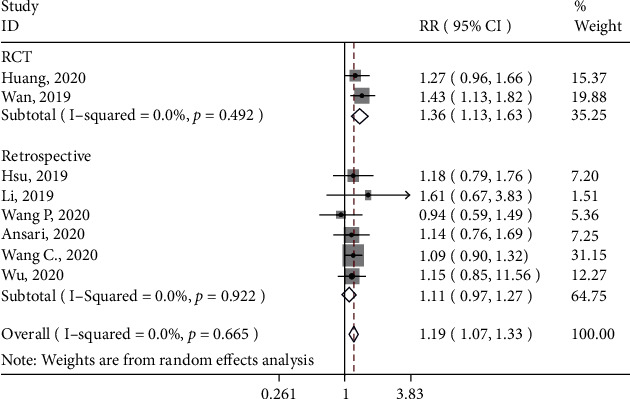
Forest plot for rate of complications. Subgroup-RCT: RR (95%CI) = 1.36 (1.34, 1.63), *z* = 3.34, *p* = 0.001; subgroup-retrospective: RR (95%CI) = 1.12 (0.97, 1.27), *z* = 1.57, *p* = 0.117; overall: RR (95%CI) = 1.19 (1.07, 1.33), *z* = 3.24, *p* = 0.001.

Eight studies assessed the rate of complications in both treatment groups. They were included in the meta-analysis. Based on the forest plot (Figure 7), overall, the control group was more likely to have complications than the 3D printing (RR: 1.19; 95% CI: 1.07, 1.33). Based on the subgroup analysis, when only findings from RCTs were included, the control group was more likely to have postsurgery complications (RR: 1.36; 95% CI: 1.34, 1.63). When findings from retrospective studies were included, the control group was more likely to have complications than the 3D printing group, but the effect was not significant (RR: 1.12; 95% CI: 0.97, 1.27).

## 4. Discussion

This systematic review analyzed the general use of 3D printing technology for the whole perioperative management including preoperative planning and intraoperative setting in the treatment of acetabular fractures. Overall, 3D printing-assisted surgery resulted in better surgery-related and clinical outcomes as compared with conventional surgery. These findings demonstrated the potential benefits of 3D printing technology in orthopedics surgery and provided further evidence for more effective management of acetabular fractures.

Acetabular fractures have brought huge physical, psychological, and functional burdens to the patients. Acetabular anatomy is complex, adjacent to important blood vessels and nerves [1]. Thus, the anatomical reconstruction of acetabular fractures is very challenging, especially for patients with complex acetabular fractures. Effective reduction and internal fixation are key to the treatment of acetabular fractures [46]. Preoperative imaging of the location and degree of acetabular fractures should be considered in order to effectively restore the biomechanical stability of the acetabulum. Similarly, the surgical approach and internal fixation method should be determined before the surgery to achieve satisfactory outcomes [19]. In recent years, 3D printing technology has been widely used for a variety of fractures. It requires comprehensive and efficient interactions between medical engineers and medical staff [47, 48]. Nonetheless, compared to traditional surgery, 3D printing-assisted surgery has unique advantages. This method could produce solid fracture models equivalent to the actual ones. The tactile feedback from those models allows the surgeons to feel resistance, contours, textures, and edges of the fractures [49, 50]. This technology can facilitate preoperative planning and thus may help to achieve accurate reduction and improve surgical outcomes.

We found that 3D technology-assisted surgery significantly improved surgery-related outcomes including operation time, intraoperative blood loss, and time of intraoperative fluoroscopy. In this review, 3D printing technology was mainly used for preoperative planning. It reduced operation time by approximately 40 minutes, intraoperative blood loss by around 260 ml, and intraoperative instrumentation time by 34 minutes. It also decreased the number/time of intraoperative fluoroscopy. These findings are consistent with the ones from previous reviews conducted in patients with other fractures (e.g., humeral fractures, elbow fractures, and pelvic fractures) [51–55]. Based on a meta-analysis of studies conducted in patients with traumatic fractures, 3D printing-assisted surgery significantly reduced operation time, intraoperative blood loss, and the number of fluoroscopies [56]. Similar results were found for patients with tibial plateau fractures [25]. Previous evidences [46, 57] suggest that 3D printing technology is reliable and accurate in the classification of acetabular fractures. Compared with conventional surgery, 3D printing technology could help the surgeons to better understand the anatomic features of the fracture and get better prepared before the surgery. During conventional surgery, important structures may be damaged and thus increase the operation time and blood loss. With the assistance of 3D printing technology, the surgeons can select suitable plates in vitro and predetermine the best position and contour of the plates [58, 59]. It thus can decrease the operation time by avoiding repeatedly bending the plates during the surgery. The surgery for complex acetabular fractures is even more difficult and with higher risks than that for simple acetabular fractures. By using 3D printing technology to simulate the operation process, the surgeons could fully understand the complex procedures and practice before the surgery, thereby improving their operation skills and decreasing the operation time [60]. Collectively, the above results suggest that 3D printing technology can optimize the operation process and improve intraoperative related outcomes.

In this review, 3D printing technology achieved a better quality of reduction and function of the hip joint than traditional surgery. These findings are in line with a previous study conducted on patients with pelvic fractures [54]. However, based on two previous meta-analyses, 3D printing technology-assisted surgery did not have a significant impact on the quality of reduction and postoperative function recovery [25, 56]. Several reasons may explain the inconsistency. In one review [56], various traumatic fractures were included such as anterior pelvic ring fracture, proximal humeral fracture, and acetabulum fracture, contributing to the significant heterogeneity between studies. In the other review [25], only tibial plateau fractures were included. Different pathophysiology is involved in different types of fractures. Acetabular fractures are more complex than tibial plateau fractures and thus require more preparations and clinical experiences. This finding suggests that patients with acetabular fractures might benefit more from 3D technology than those with simple fractures.

In this review, we also found that 3D printing technology reduced postoperative complications. This finding is in line with the one from a previous systematic review conducted in patients with mandibular angle fractures [61]. Acetabular fractures are intra-articular fractures, and most acetabular fractures have a complex three-dimensional displacement (i.e., rotational displacement). The surgery of acetabular fractures is large; achieving anatomical reduction and firm fixation as well as preventing the surgical and postsurgery complications are key to the success of its treatment [46]. There are several vital steps during the surgery, including protecting the blood supply of the sciatic nerve and femoral head, protecting the L5 nerve root and femoral nerve, and reducing the risk of heterotopic ossification [62, 63]. In traditional surgery without the assistance of 3D printing technology, surgeons might be limited by factors (e.g., angle of the fracture site or overlapping fracture patches), which may often lead to uneven joint surface, resulting in a high incidence of postoperative complications [19]. With 3D printing technology, a fracture model of the patient can be produced before the surgery. The surgeons thus can have a more intuitive understanding of the fracture characteristics, develop an individualized therapeutic regimen, and simulate the surgery process. Those advantages could improve the clinical effect and postoperative functions of the patients [42].

In recent years, 3D printing technology has been widely used in orthopedics surgery. To the best of our knowledge, the systematic review was among the first that quantified the efficacy of using 3D technology in the treatment of acetabular fractures. However, findings from this review need to be interpreted in light of the limitations. A majority of the studies included in this review were conducted in China. Findings from this review may not be generalized to western countries. Similarly, patients included in this review were mostly middle-aged. The rate of acetabular fractures in the elderly is on the rise, with a rise of up to 23% per annum [64]. Management of acetabular fractures in the elderly requires a unique approach, due to complexities conferred by underlying conditions and compromised bone quality [65]. Thus, studies focusing on the geriatric population are warranted. In addition, this review analyzed the general use of 3D printing technology for the whole perioperative management, and data were pooled together, which has precluded us from looking at the separate efficacy of 3D printing technology for preoperative planning and intraoperative setting. Studies included in this review were mainly retrospective in design, limiting the causal inference. We performed subgroup analyses based on study design, and most of the findings were robust. With the increasing use of 3D printing technology, there will be more studies investigating the safety and efficacy of its use. Future studies should consider using a more rigorous design (e.g., randomized trial with blinding). Another limitation is the lack of follow-up information. Although postoperation complications were measured, the long-term benefits of 3D printing technology could be assessed. Some complications may occur after a long period of time (e.g., secondary osteoarthritis). Thus, a long-term follow-up is needed in future research to provide further evidence for the clinical use of 3D printing technology in the treatment of fractures. In this review, few studies examined the time used for preoperative planning (e.g., printing time). Recent evidence suggests the benefits of developing a 3D printing workflow [66, 67]. As such, more studies of this type are warranted.

In conclusion, 3D printing technology demonstrated efficacy in the treatment of acetabular fractures. It may reduce operation time, blood loss, and postoperative complications as well as improve the quality of reduction and function of the hip joint. Nonetheless, surgeons should bear in mind that the use of 3D printing technology requires them not only to have rich clinical experience in preoperative design but also to master the application of digital orthopedic software. Although there is an initial learning curve, these become easier with practice and experience. In addition, high-quality CT images are required to improve the accuracy of the simulating model and avoid errors during the surgery. 3D printing technology also has limitations such as not being able to reflect the blood vessels, nerves, and other conditions of the bone injury site. The guidance of experienced surgeons is thus needed.

## Figures and Tables

**Table:** (a) Risk of bias in randomized controlled trials

First author, year	Selection bias		Performance bias	Detection bias	Attrition bias	Reporting bias
	Randomization	Concealment	Participant/personnel blinding	Assessor blinding		
Huang, 2020 [37]	L	UC	H	L^∗^	L	L
Maini, 2018 [39]	L	UC	H	L^∗^	L	L
Maini, 2018 [40]	L	UC	H	L^∗^	L	H
Wan, 2019 [42]	L	UC	H	L^∗^	L	L

L: low risk; H: high risk; UC: unclear; ^∗^it was not clear whether the assessor was blinded; however, the outcome measures were mainly objective and unlikely to be influenced by knowledge of the intervention received by the participants.

**Table:** (b) Risk of bias in retrospective comparative studies

First author, year	Selection	Comparability	Exposure/outcome
Chen, 2019 [34]	★★★★	★	★★★
Hsu, 2019 [36]	★★★★	★	★★★
Li, 2019 [38]	★★★★	★	★★★
Wang, 2020 [44]	★★★★	★	★★★
Ansari, 2020 [33]	★★★★	★	★★★
Downey, 2020 [35]	★★★★	★	★★★
Öztürk, 2020 [41]	★★★★	★	★★★
Wang, 2020 [43]	★★★★	★	★★★
Wu, 2020 [45]	★★★★	★	★★★

**Table 2:** Characteristics of included studies (*N* = 13).

Study (1^st^ author, year, country)	Design	Sample size	Notes on inclusion or exclusion criteria	Sample characteristics (age, sex, and other parameters)	3D printing technology used and software	Outcomes and follow-ups
Ansari, 2020, India [33]	Retrospective comparative	Group E: 12 Group C: 15	Inclusion: acetabular fracture, time from injury to surgery < 2 weeks Exclusion: simple wall fractures, periprosthetic fractures	Age (years): Group E: 41.3 (13.7) Group C: 39.1 (12.4) Male: Group E: 11 (92%) Group C: 12 (80%) Time from injury to surgery (days): Group E: 9.3 (2.8) Group C: 8.3 (2.4)	3D-printed model for preoperative planning (precontoured plates) 3D image processing using syngo via VB40 software (Siemens, Munich, Germany) and printing using Da-Vinci 1.0A printer (XYZ printing, Taiwan)	Surgery-related outcomes: operation time, blood loss, quality of reduction (Matta scoring system), number of intraoperative fluoroscopy; function of hip joint (Harris hip score); complications
Chen, 2019, China [34]	Retrospective comparative	Group E: 28 Group C: 24	Inclusion: closed complex acetabular fracture involving two columns with a history of surgical treatment Exclusion: inability to undergo surgery within 3 weeks of injury	Age (years): Group E: 46.1 (13.6) Group C: 42.4 (12.3) Male: Group E: 18 (64%) Group C: 14 (58%) Time from injury to surgery: 9.4 (4.2) days, ranging from 4 to 21 days	3D-printed model for preoperative planning (precontoured plates) 3D image processing software MIMICS 16.0 (Materialise, Belgium) and Cura (Ultimaker, Netherlands)	Surgery-related outcomes: operation time, blood loss, time required to contour plates during the operation, quality of reduction (Matta scoring system); function of hip joint (Merle d'Aubigné scores)
Downey, 2020, Ireland [35]	Retrospective comparative	Group E: 10 Group C: 10	Inclusion: complex pelvic and acetabular fractures.	Age (years): Group E: 51.9 (18.9) Group C: 51.8 (14.9) Male: Group E: 9 (90%) Group C: 9 (90%)	3D-printed model for preoperative planning (precontoured plates) Meshmixer software	Surgery-related outcomes: operation time, blood loss, radiation exposure; postinjury health status
Hsu, 2019, Taiwan (China) [36]	Retrospective comparative	Group E: 12 Group C: 17	Exclusion: acetabular fractures treated with column screw fixation	Age (years): Group E: 36.8 (16.4) Group C: 38.2 (16.4) Male: Group E: 11 (92%) Group C: 14 (82%) BMI (kg/m^2^): Group E: 26.2 (6.1) Group C: 25.8 (3.4)	3D-printed model for preoperative planning (precontoured plates) 3D image processing software MIMICS 19.0 (Materialise, Belgium) and 3D modeling desktop machinery (UP BOX+, Tiertime, China, or Mass Portal XD 40, Mass Portal, Latvia)	Surgery-related outcomes: operation time, instrumentation time, blood loss, quality of reduction (a displacement of <2 mm was considered good); complications
Huang, 2020, China [37]	Prospective RCT	Group E: 20 Group C: 20	Inclusion: both-column acetabular fractures and a lapse of <3 m from injury	Age (years): Group E: 43.4 (11.6) Group C: 37.4 (12.7) Male: Group E: 12 (60%) Group C: 14 (70%) Time from injury to surgery (days): Group E: 9.2 (3.8) Group C: 8.8 (3.7)	3D-printed model for preoperative planning (precontoured plates) 3D image processing software MIMICS 15.0 (Materialise, Belgium) and 3D printer (Prismlab Rapid400; Prismlab, Shanghai, China)	Surgery-related outcomes: operation time, blood loss, instrumentation time, time of intraoperative fluoroscopy, quality of reduction (a displacement of <2 mm was considered good); function of hip joint (Harris score); complications
Li, 2019, Taiwan (China) [38]	Retrospective comparative	Group E: 7 Group C: 9	Inclusion: traumatic dislocation of the hip joint combined with acetabular fractures Exclusion: acetabular fractures combined with pelvic iliac wing fractures	Age (years): Group E: 32.1 (14.6) Group C: 37.0 (17.1) Male: Group E: 7 (100%) Group C: 6 (66.7%) BMI (kg/m^2^): Group E: 26.3 (2.3) Group C: 27.2 (3.0)	3D-printed model for preoperative planning (precontoured plates) 3D image processing software MIMICS 19.0 (Materialise, Belgium) and 3D modeling desktop machinery (UP BOX+, Tiertime, China, or Mass Portal XD 40, Mass Portal, Latvia)	Surgery-related outcomes: operation time, instrumentation time, blood loss, quality of reduction (a displacement of <2 mm was considered good); complications
Maini, 2018, India [39]	Prospective RCT	Group E: 10 Group C: 11	Inclusion: displaced acetabulum fractures with displacement of over 3 mm within 3 weeks of injury	Age (years): Group E: 25-59 Group C: 18-60 Male: Group E: 9 (90%) Group C: 9 (82%)	3D-printed model for preoperative planning (precontoured plates) 3D image processing software MIMICS 8.13 (Materialise, Belgium) and 3D printer EOSINT P380 (EOS, Birmingham, UK)	Surgery-related outcomes: operation time, blood loss, quality of reduction (Matta scoring system)
Maini and Verma, 2018, India [40]	Prospective RCT	Group E: 12 Group C: 13	Same as above	Age (years): Group E: 25-72 Group C: 22-65 Male: Group E: 11 (92%) Group C: 12 (92%)	Same as above	Same as above
Öztürk, 2020, Turkey [41]	Retrospective comparative	Group E: 9 Group C: 9	Inclusion: unilateral acetabular fracture Exclusion: previous acetabular surgery	Age (years): Group E: 46.2 (30-66) Group C: 41.7 (16-70) Male: Group E: 9 (100%) Group C: 9 (100%)	3D-printed model for preoperative planning (precontoured plates) Discovery St PET/CT scanner (General Electric, Milwaukee, WI, USA) and 3D printer (Formlabs Inc. 35 Medford St. Suite 201, Somerville, MA, USA)	Surgery-related outcomes: operation time, blood loss, instrumentation time, number of intraoperative fluoroscopy, quality of reduction (a displacement of <2 mm was considered good)
Wan, 2019, China [42]	Prospective RCT	Group E: 48 Group C: 48	Inclusion: complex hip fracture and fracture of acetabular posterior wall, within 2 weeks of injury	Age (years): Group E: 43.4 (4.5) Group C: 41.9 (5.0) Male: Group E: 34 (71%) Group C: 32 (67%) Time from injury to surgery (days): Group E: 10.1 (1.4) Group C: 10.4 (1.1)	3D-printed model for preoperative planning (precontoured plates) 3D image processing software MIMICS 14.0 (Materialise, Belgium) and MakerBot Replicator 2 printer	Surgery-related outcomes: operation time, blood loss, number of intraoperative fluoroscopy, quality of reduction (Matta scoring system); function of hip joint (Harris score); complications
Wang, 2020, China [43]	Retrospective comparative	Group E: 15 Group C: 35	Inclusion: acute (<21 days) and unilateral acetabular fractures Exclusion: open fractures of acetabulum	Age (years): Group E: 46.6 (12.3) Group C: 45.1 (12.6) Male: Group E: 10 (67%) Group C: 22 (63%) Time from injury to surgery (days): Group E: 8.6 (3.0) Group C: 8.1 (4.1)	3D-printed plates used during surgery 3D image processing using Mimics 15.0 software (Materialise, Leuven, Belgium) and printing using selective laser melting (SLM) 3D printer (DiMetal-100, SCUT, Guangzhou, China)	Surgery-related outcomes: operation time, blood loss, instrumentation time, times of intraoperative fluoroscopy, quality of reduction (Matta scoring system); complications
Wang, 2020, China [44]	Retrospective comparative	Group E: 41 Group C: 47	Inclusion: complex acetabular fractures, time from injury <3 weeks	Age (years): Group E: 46.8 (12.0) Group C: 41.5 (11.7) Male: Group E: 29 (71%) Group C: 32 (68%) BMI (kg/m^2^): Group E: 26.3 (2.3) Group C: 27.2 (3.0)	3D-printed model for preoperative planning (precontoured plates) 3D image processing software MIMICS (Materialise, Belgium) and 3D printer (MakerBot Replicator, NY, USA)	Surgery-related outcomes: operation time, blood loss, quality of reduction (Matta scoring system); function of hip joint (Modified Postel Merle D'Aubigné score); complications
Wu, 2020, China [45]	Retrospective comparative	Group E: 20 Group C: 23	Inclusion: displaced double-column acetabular fractures Exclusion: time from injury to surgery > 3 weeks	Age (years): Group E: 50.1 (8.2) Group C: 51.0 (8.6) Male: Group E: 15 (75%) Group C: 16 (70%) Time from injury to surgery (days): Group E: 9.0 (3.0) Group C: 9.2 (2.8)	3D-printed plates used during surgery 3D image processing using Mimics 20.0 software (Materialise, Leuven, Belgium) and printing using Huasen Medical Instruments	Surgery-related outcomes: operation time, blood loss, quality of reduction (Matta scoring system); function of hip joint (Modified Postel Merle D'Aubigné score); complications

Group E: experimental group receiving 3D printing assisted surgery; Group C: control group receiving traditional surgery; BMI: body mass index; RCT: randomized controlled trial.

**Table 3:** Main outcomes from each study (*N* = 13).

Study (1^st^ author, year, country)	Operation time (minutes)	Intraoperative blood loss (ml)	Quality of reduction (rate of good/excellent)	Other clinical outcomes	Complications
Ansari, 2020, India [33]	Group E: 184 (39) Group C: 246 (44)	Group E: 664 (186) Group C: 936 (198)	Group E: 11 (91.7%) Group C: 11 (73.3%)	Function of hip joint (score): Group E: 79.7 (13.7) Group C: 83.4 (12.3); *p* = 0.23 Number of intraoperative fluoroscopy: Group E: 22.0 (5.6) Group C: 62.0 (16.5); *p* < 0.05	Rate: Group E: 2 (16.7%) Group C: 4 (26.7%) Included: surgical site infection (in both groups), sciatic nerve palsy, and avascular necrosis of femoral head
Chen, 2019, China [34]	Group E: 158 (20) Group C: 187 (36)	Group E: 696 (167) Group C: 834 (227)	Group E: 25 (89.3%) Group C: 19 (79.2%)	Function of hip joint-rate of excellent/good: Group E: 24 (85.7%) Group C: 18 (75.0%)	/
Downey, 2020, Ireland [35]	Group E: 130 (62) Group C: 122 (57)	Group E: 825 (724) Group C: 690 (583)	/	Radiation exposure (mGy/cm^2^): Group E: 1078 (800) Group C: 727 (349); *p* > 0.05 No group difference in postsurgery healthy status (e.g., mobility, self-care, and depression)	
Hsu, 2019, Taiwan (China) [36]	Group E: 199 (50) Group C: 274 (81)	Group E: 433 (317) Group C: 958 (427)	Group E: 11 (91.7%) Group C: 14 (82.4%)	Instrumentation time (minutes): Group E: 43 (11) Group C: 99 (15)	Rate: Group E: 2 (16.7%) Group C: 5 (29.4%) Included: Group E: avascular necrosis of femoral head and superior gluteal artery injury Group C: heterotopic ossification, sciatic nerve injury, traumatic arthritis
Huang, 2020, China [37]	Group E: 131 (29) Group C: 206 (35)	Group E: 500 (400: 800) Group C: 1050 (950: 1200)	Group E: 16 (80%) Group C: 6 (30%)	Instrumentation time (minutes): Group E: 32 (10) Group C: 58 (15) Time of intraoperative fluoroscopy (seconds): Group E: 4.2 (1.8) Group C: 7.7 (2.6); *p* < 0.001 Function of hip joint-rate of excellent/good: Group E: 15 (75%) Group C: 6 (30%)	Rate: Group E: 1 (5%) Group C: 5 (25%) Included: Group E: heterotopic ossification 2 months after the operation Group C: inflammatory response, heterotopic ossification, iatrogenic neurological symptoms, traumatic arthritis
Li, 2019, Taiwan (China) [38]	Group E: 212 (52) Group C: 254 (34)	Group E: 736 (614) Group C: 742 (229)	Group E: 7 (100%) Group C: 7 (77.8%)	Instrumentation time (minutes): Group E: 38 (11) Group C: 72 (10)	Rate: Group E: 2 (28.6%) Group C: 5 (55.6%) Included: Case: avascular necrosis of femoral head and superior gluteal artery injury Control: avascular necrosis of femoral head, heterotopic ossification, and traumatic arthritis
Maini, 2018, India [39]	Group E: 32 (41) Group C: 120 (38)	Group E: 620 (247) Group C: 720 (286)	Group E: 8 (80%) Group C: 7 (64%)	/	/
Maini, 2018, India [40]	Group E: 111 (38)^∗^ Group C: 119 (31)^∗^	Group E: 467 (129)^∗^ Group C: 525 (211)^∗^	Group E: 9 (75%) Group C: 8 (61%)		
Öztürk, 2020, Turkey [41]	Group E: 181 (9) Group C: 200 (16)	Group E: 403 (53) Group C: 607 (53)	Group E: 8 (89%) Group C: 6 (78%)	Instrumentation time (minutes): Group E: 36 (4) Group C: 57 (11) Number of intraoperative fluoroscopy: Group E: 6.0 (0.9) Group C: 10.4 (2.2); *p* < 0.05	No serious complications during follow-up
Wan, 2019, China [42]	Group E: 211 (55) Group C: 296 (66)	Group E: 1147 (235) Group C: 1832 (268)	Group E: 39 (81.3%) Group C: 37 (77.1%)	Number of intraoperative fluoroscopy: Group E: 6.8 (1.6) Group C: 12.4 (2.1); *p* < 0.001 Function of hip joint-rate of excellent/good: Group E: 42 (87.5%) Group C: 40 (83.3%)	Rate: Group E: 5 (10.4%) Group C: 18 (37.5%) Included: Inflammatory response, ectopic ossification, iatrogenic neurological symptoms, traumatic arthritis
Wang, 2020, China [43]	Group E: 142 (53) Group C: 171 (41)	Group E: 880 (673) Group C: 1177 (692)	Group E: 14 (93.3%) Group C: 31 (88.6%)	/	Rate: Group E: 1 (6.7%) Group C: 5 (14.3%) Included: Loose of public screw (Group E), surgical site infection, DVT, traumatic arthritis, obturator nerve injury
Wang, 2020, China [44]	Group E: 212 (63) Group C: 264 (56)	Group E: 654 (126) Group C: 874 (536)	Group E: 35 (85.4%) Group C: 38 (78.8%)	Function of hip joint-rate of excellent/good: Group E: 34 (83.0%) Group C: 33 (70.2%)	Rate: Group E: 23 (56.1%) Group C: 25 (44.6%) Included: Venous thromboembolism, lateral femoral cutaneous nerve injury, infection, arthritis, avascular necrosis, heterotopic ossification
Wu, 2020, China [45]	Group E: 223 (45) Group C: 261 (57)	Group E: 930 (523) Group C: 1426 (733)	Group E: 18 (90%) Group C: 20 (87%)	Function of hip joint-rate of excellent/good: Group E: 18 (90%) Group C: 19 (83%)	Rate: Group E: 3 (15%) Group C: 6 (26%) Included: Traumatic arthritis and lateral femoral cutaneous nerve injury (Group E); DVT, traumatic arthritis, and lateral femoral cutaneous nerve injury (Group C)

Group E: experimental group receiving 3D printing assisted surgery; Group C: control group receiving traditional surgery; ^∗^standard deviation was calculated based on available information.

## Data Availability

Data will be available upon request.
